# Technical considerations in PCR-based assay design for diagnostic DNA methylation cancer biomarkers

**DOI:** 10.1186/s13148-022-01273-z

**Published:** 2022-04-27

**Authors:** Maartje Massen, Kim Lommen, Kim A. D. Wouters, Johan Vandersmissen, Wim van Criekinge, James G. Herman, Veerle Melotte, Leo J. Schouten, Manon van Engeland, Kim M. Smits

**Affiliations:** 1 Department of Pathology, GROW – School for Oncology and Reproduction, Maastricht University Medical Center, P.O. Box 616, 6200 MD Maastricht, The Netherlands; 2 MDxHealth, Inc., Irvine, CA 92618 USA; 3 Department of Mathematical Modelling, Statistics and Bioinformatics, Ghent University, 9000 Ghent, Belgium; 4 The Hillman Cancer Center, University of Pittsburgh Cancer Institute, Pittsburgh, PA 15232 USA; 5 Department of Clinical Genetics, Erasmus University Medical Center, 3015 GD Rotterdam, The Netherlands; 6 Department of Epidemiology, GROW – School for Oncology and Reproduction, Maastricht University Medical Center, P.O. Box 616, 6200 MD Maastricht, The Netherlands

**Keywords:** Epigenetics, DNA methylation, Diagnosis, Biomarkers, Assay design, Genomic location, Cancer biomarkers

## Abstract

**Background:**

DNA methylation biomarkers for early detection, risk stratification and treatment response in cancer have been of great interest over the past decades. Nevertheless, clinical implementation of these biomarkers is limited, as only < 1% of the identified biomarkers is translated into a clinical or commercial setting. Technical factors such as a suboptimal genomic location of the assay and inefficient primer or probe design have been emphasized as important pitfalls in biomarker research. Here, we use eleven diagnostic DNA methylation biomarkers for colorectal cancer (*ALX4*,* APC*,* CDKN2A*,* MGMT*,* MLH1*,* NDRG4*,* SDC2*,* SFRP1*,* SFRP2*,* TFPI1* and *VIM*), previously described in a systematic literature search, to evaluate these pitfalls.

**Results:**

To assess the genomic assay location, the optimal genomic locations according to TCGA data were extracted and compared to the genomic locations used in the published assays for all eleven biomarkers. In addition, all primers and probes were technically evaluated according to several criteria, based on literature and expert opinion. Both assay location and assay design quality varied widely among studies.

**Conclusions:**

Large variation in both assay location and design hinders the development of future DNA methylation biomarkers as well as inter-study comparability.

## Background

DNA methylation biomarkers for early detection, risk stratification and treatment response have been of great interest in the clinical management of cancer. Over the past decades, the focus in DNA methylation biomarkers research has expanded from tissue to liquid biopsies. Since then, some of these biomarkers have been incorporated in commercially available diagnostic tests [1]. In a recent systematic literature review, 100 potentially published DNA methylation biomarkers for colorectal cancer (CRC) were identified in bodily fluids (Feng et al*.* unpublished data). Only three of these (*NDRG4*, *BMP3* and *SEPT9*) have been translated into commercial tests currently available for the early detection of CRC [1]. Various reasons for this suboptimal clinical translation have been postulated [1–3]; many of these focus on issues such as a suboptimal study design, lack of validation and lack of clinical relevance. However, technical factors such as a suboptimal genomic location of the assay and inefficient primer or probe design have been emphasized as important pitfalls in biomarker research as well [1, 3–5]. The choice of which genomic location to study in the evaluation of DNA methylation biomarkers can influence the conclusion on the clinical value of this biomarker. Koch et al*.* previously described the importance of selecting the optimal genomic location, for example, by using publicly available data such as The Cancer Genome Atlas (TCGA) or whole-genome sequencing data [1, 6]. These data can be used to identify the genomic location with the largest methylation differences between sample groups, associated with the clinical outcome of interest. For example, we assume that the genomic locations with the largest difference in methylation between normal and tumor samples can be used to discriminate tumor tissue/patients from normal tissue/healthy individuals, as suggested in several of our previous publications [1, 7, 8].

In addition to the identification of these extracted locations with the largest difference between normal and tumor tissue, several technical assay design issues are crucial for optimal DNA methylation biomarker development and subsequent chances for successful clinical translation, including assay type and primer- and probe design. For DNA methylation analysis, the most widely used technique is (quantitative) methylation-specific PCR (MSP/qMSP), which requires primer and probe design on the bisulfite-converted sequence of the biomarker of interest [9]. Although MSP primer design tools (including Bisearch, Methprimer and PrimerSuite) are available [10], these tools do not incorporate publicly available genomic data, and therefore do not preselect the most optimal genomic region for assay design.

Here, we analyzed the diagnostic CRC methylation biomarkers identified in a previously conducted systematic literature search in order to provide an overview of the genomic locations. Moreover, we evaluated the quality of the described primers and probes and define recommendations that can guide assay design within the DNA methylation biomarker field.

## Results and discussion

### Dataset characteristics

**Fig. 1:**
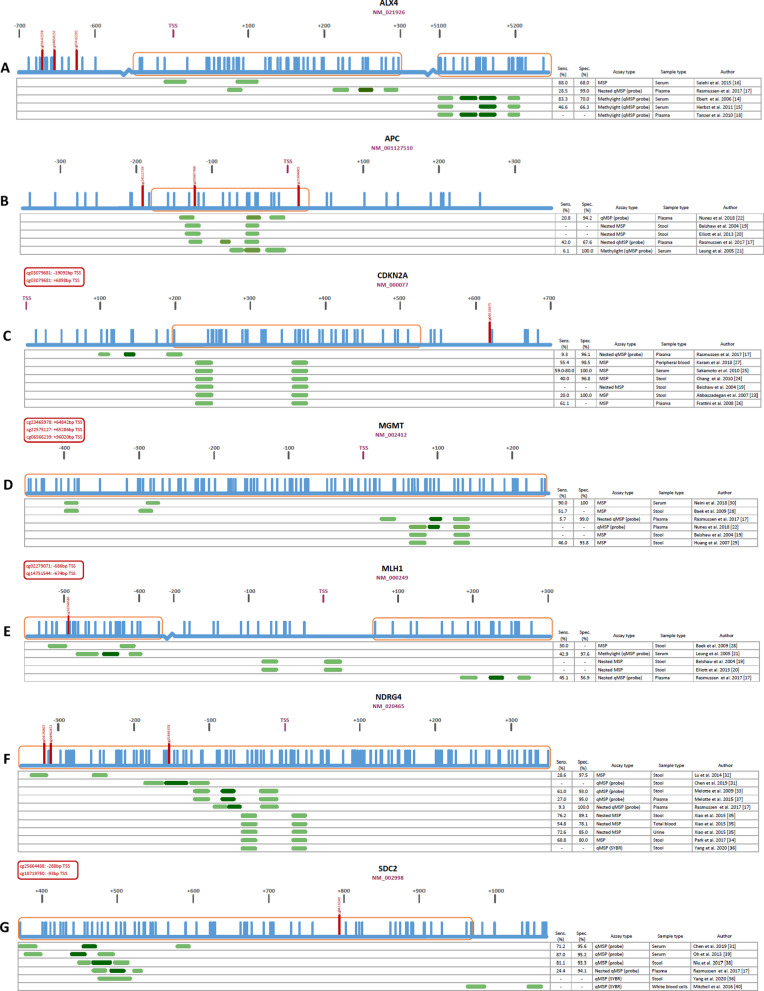

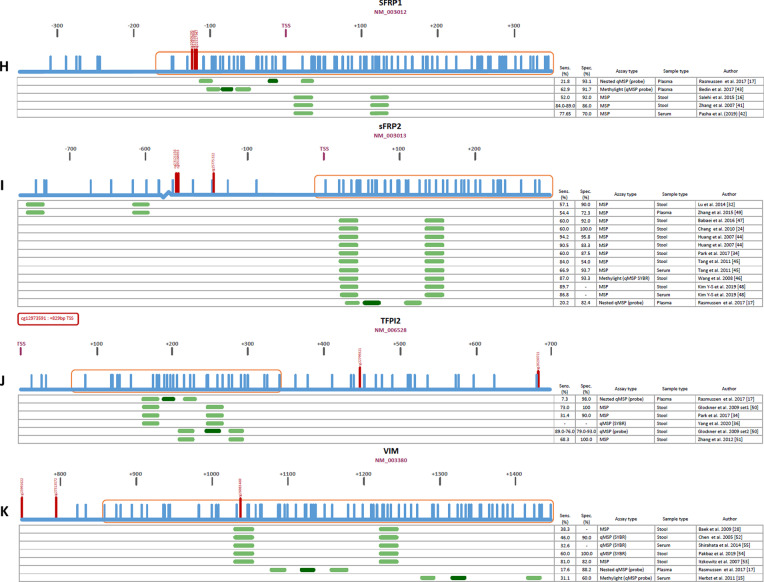
**A**–**K** Genomic locations, extracted CG’s (from TCGA) and diagnostic performances of the investigated assays per marker.  : CpG islands,  : CGs,  : extracted CGs (obtained from TCGA),  : primers,  : probes, TSS: transcription start site, Sens: sensitivity, Spec: specificity

Here, we provided an overview of the studied genomic locations, the extracted locations according to TCGA data, and the quality of used primers and probes for the 11 most studied diagnostic DNA methylation biomarkers in CRC (*ALX4*,* APC*,* CDKN2A*,* MGMT*,* MLH1*,* NDRG4*,* SDC2*,* SFRP1*,* SFRP2*,* TFPI2*,* VIM)*. All genes were evaluated in a minimum of five (*TFPI2*) and a maximum of 12 (*SFRP2*) independent studies (Table 1). Markers had been studied in a variety of bodily fluids including stool, serum, plasma and urine. Diagnostic performance (sensitivity and specificity) showed considerable variation between individual studies evaluating the same marker, which might be attributed to sample type differences. *MGMT* showed the largest sensitivity range of 5.7–90.0% across sample types, with specificities varying from 93.8 to 100%. *MLH1* showed the smallest range in sensitivity (30.0–45.1%); however, the specificity range was substantial (56.9–97.6%). Despite using identical assays, diagnostic performance of these studies varied widely; e.g., 20–80% sensitivity and 96.8–100% specificity for *CDKN2A*, 60–94.2% sensitivity and 54–100% specificity for *SFRP2* and 32.6–81% sensitivity and 82–100% specificity for *VIM* (Fig. 1C, I, K). This might be attributed to sample type differences, as illustrated by the relatively low sensitivities of *CDKN2A* methylation in stool (20–40%), compared to serum (59–80%), plasma (61.1%) and peripheral blood (55.4%) using the same assay (Table 1; Fig. 1C). Similarly, the diagnostic performance of *sFRP2* in stool varied more widely (sensitivity 60–94.2%, specificity 54–100%) compared to serum (sensitivity 66.9–86.8%, specificity 93.7%) using the same assay (Table 1; Fig. 1I). As stool contains PCR inhibitors like complex polysaccharides and bile salts, undigested debris, and an abundance of, e.g., bacterial DNA over human DNA, this can explain the lack of performance in these samples, compared to blood-based samples [11]. In addition, plasma seems to perform worse compared to serum using the same assay for most markers (Table 1; Fig. 1), which is in line with literature, suggesting that DNA is more abundant and stable in serum compared to plasma [12, 13]. On the other hand, the diagnostic performances within one gene using the same sample type but different also differs widely, as illustrated by the sensitivities of, e.g., sFRP1 in plasma (Rasmussen et al*.* 21.8%; Bedin et al*.* 62.9%), CDKN2A in plasma (Rasmussen et al*.* 9.3%; Frattini et al*.* 61.1%) and *NDRG4* in stool (Lu et al*.* 28.6%; Melotte et al*.* 61.0%; Xiao et al*.* and Park et al*.* 68.8–76.2%). Therefore, it seems that the performance of these biomarkers is influenced by both sample type and assay.

**Table 1:** Summary of the 11 most studied DNA methylation biomarkers for CRC in liquid biopsies

Gene	Number of articles	Liquid biopsy type	Sensitivity range	Specificity range
*ALX4*	3 [14–16]	Serum	46.6–88.0%	66.3–70.0%
	2 [17, 18]	Plasma	28.5–80.0%	41.0–99.0%
*APC*	2 [19, 20]	Stool	-	-
	1 [21]	Serum	6.1%	100%
	2 [17, 22]	Plasma	20.8–42-0%	67.6–94.2%
*CDKN2A*	3 [19, 23, 24]	Stool	20.0–40.0%	96.8–100%
	1 [25]	Serum	59.0–80.0	100%
	2 [17, 26]	Plasma	9.3–61.1%	96.1%
	1 [27]	Peripheral blood	55.4%	98.5%
*MGMT*	3 [19, 28, 29]	Stool	46.0–51.7%	93.8%
	1 [30]	Serum	90.0%	100%
	2 [17, 22]	Plasma	5.7%	99.0%
*MLH1*	3 [19, 20, 28]	Stool	30.0%	-
	1 [21]	Serum	42.9%	97.6%
	1 [17]	Plasma	45.1%	46.9%
*NDRG4*	6 [31–36]	Stool	28.6–76.2%	80.0–97.5%
	2 [17, 37]	Plasma	9.3–27.0%	95.0–100%
	1 [35]	Total blood	54.8%	78.1%
	1 [35]	Urine	72.6%	85.0%
*SDC2*	2 [36, 38]	Stool	81.1%	93.3%
	2 [31, 39]	Serum	71.2–87.0%	95.2–95.6%
	1 [17]	Plasma	24.4%	94.1%
	1 [40]	White blood cells	-	-
*sFRP1*	2 [16, 41]	Stool	52.0–89.0%	86.0–92.0%
	1 [42]	Serum	77.7%	70.0%
	2 [17, 43]	Plasma	21.8–62.9%	91.7–93.1%
*sFRP2*	9 [24, 29, 32, 34, 44–48]	Stool	57.1–94.2%	54.0–100%
	2 [45, 48]	Serum	66.9–86.8%	93.7%
	2 [17, 49]	Plasma	20.2–54.4%	72.3–82.4%
*TFPI2*	4 [34, 36, 50, 51]	Stool	31.4–89.0%	79.0–100%
	1 [17]	Plasma	7.3%	98.0%
*VIM*	4 [28, 52–54]	Stool	38.3–81.0%	82.0–100%
	2 [15, 55]	Serum	31.1–32.6%	60%
	1 [17]	Plasma	17.6%	88.2%

### Overview of genomic and extracted locations of selected assays

In the 11 most studied diagnostic DNA methylation biomarkers for CRC, multiple genomic locations were studied. An overview of all genomic locations in the individual studies is presented in Fig. 1. In addition, the extracted locations that we identified from TCGA data were compared to the locations used in all published assays.

For *ALX4*,* APC*,* MGMT*,* sFRP1*,* sFRP2 TFPI2* and *VIM*, the least variation in genomic locations was observed among studies (three different genomic locations; Fig. 1A, B, D, H, I, J, K). For *APC* and *VIM*, most assays (4/5 and 5/7 respectively) included at least one of the extracted locations as identified in TCGA data (Fig. 1B, K). In contrast, none of the assays investigating *ALX4*,* MGMT*,* sFRP1*,* sFRP2* and *TFPI2* included an extracted CG (Fig. 1A, D, H, I, J). Although *MLH1* and *NDRG4* were studied in four and five genomic locations respectively, most assays did not contain an extracted CG (3/4 for *MLH1*, 3/5 for *NDRG4*; Fig. 1E, F). Largest variation in genomic location was observed for *SDC2*; however, none of these included an extracted CG (Fig. 1G).

These results show that there is a large variation in the investigated genomic locations among the different assays, whereas most studies do not specify a specific rationale for their used genomic location. Next to these variations in genomic location, we also observed a large variation in diagnostic performance even within the same genes. As previously postulated, the exact studied genomic location could influence the diagnostic performance of a biomarker, emphasizing the importance of considering genomic location of the assay upfront [1, 7, 8]. Currently, to our knowledge, no guidelines for identifying the optimal genomic location for diagnostic DNA methylation biomarkers are described. However, we previously recommended using TCGA data to identify the genomic location where the difference in methylation between normal and tumor tissue is largest. In theory, these locations might represent the most clinically relevant methylation sites for diagnostic purposes. Even though TCGA is a very accessible data source, it is limited in the amount of covered CGs. TCGA data is based on Infinium 450 K microarrays, of which the probes do not necessarily cover the most relevant CGs among the genome [1]. However, all genomic regions illustrated in this overview were covered by Illumina 450 K methylation array probes according to MEXPRESS. To assure full genomic coverage, sequencing prior to deciding on the genomic location covered in the methylation assay would be required. This has not always been feasible in the past, especially for small research groups with limited funding. The decreased sequencing costs and the availability of sequencing facilities (in both academic and commercial setting) combined with publicly available DNA methylation and gene expression data now provide opportunities to identify the most optimal genomic location for a DNA methylation marker [1]. Unfortunately, these sequencing data are rarely publicly available, which did not allow us to consider these in this manuscript.

### Primer and probe assessment

**Fig. 2:**
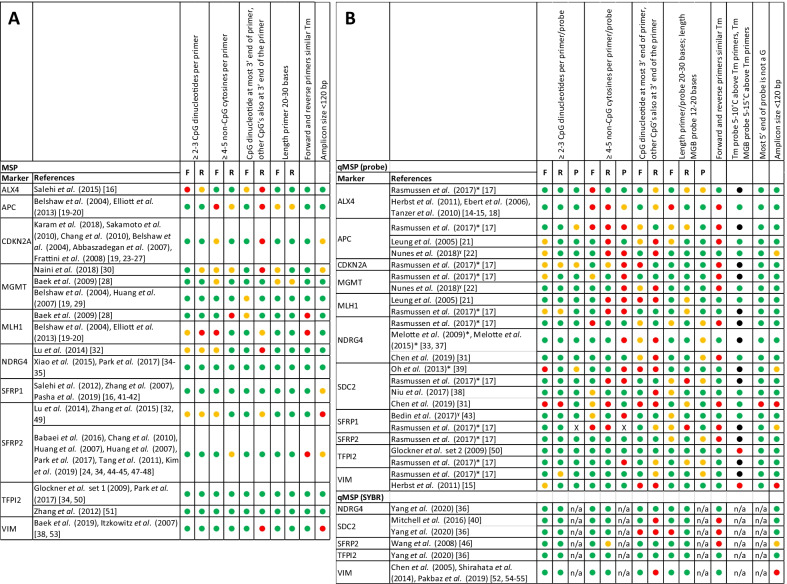
Primer and probe quality assessment of all markers and studies in MSP (**A**), qMSP assays (probe/SYBR) (**B**). F: forward primer, R: reverse primer, P: probe, n/a: not applicable, *: Molecular beacon probe, additional bases included in evaluation, ˠ: Minor groove binder probe, X: probe sequence could not be mapped back to gene.  : optimal design,  : suboptimal, but acceptable design,  : increased risk of technical problems with the primer or probe,  : not scorable because of molecular beacon probe

Of the 47 assays used to measure the 11 included markers, 16 (34%) were MSP assays, 25 (53%) were qMSP assays with probe, and 6 (13%) were qMSP assays with SYBR (Fig. 2). As bisulfite-conversion changes unmethylated cytosines to uracil, while methylated cytosines remain unchanged, the CpG dinucleotides and non-CpG cytosines in the primers define the discriminative power of the primers to distinguish methylated from unmethylated DNA [9]. As an alternative to this damaging and fragmenting bisulfite conversion, an enzymatic modification kit to enable distinguishing methylated from unmethylated DNA has become available that is less damaging to the DNA in terms of fragmentation [56]. This novel enzymatic conversion could therefore impact assay design. However, as specific issues of, e.g., bisulfite conversion have been described in detail before [57], they are not evaluated in this manuscript.

In the MSP assays, 1 forward and 1 reverse primer (6.25%) failed to include at least 2 CpG dinucleotides (Fig. 2A), whereas 2 forward primers and 1 reverse primer (4.8%) failed to include at least 2 CpG dinucleotides in the qMSP assays (Fig. 2B). All probes met this criterium (Fig. 2B). Additionally, in the MSP assays 2 forward primers and 1 reverse primer (9.4%), and 5 forward primers and 9 reverse primers (22.6%) in the qMSP assays failed to include at least 4 non-CpG cytosines (Fig. 2). Almost half of all probes (48%) in the qMSP assays with probe did not meet this criterium (Fig. 2B). Not meeting these criteria could lead to inefficient annealing and unspecific binding of the primers and probes, resulting in inconclusive findings. Inefficient annealing could result in false negatives due to the lack of amplification, even when the target sequence is available. Unspecific binding could result in false positives due to binding even when the target sequence is not fully complementary to the primer or probe [9, 58–61].

Next, 6 reverse primers (18.8%) in the MSP assays, and 6 forward and 14 reverse primers (32.2%) in the qMSP assays did not carry a CpG dinucleotide at the most 3’ end of the primer (Fig. 2). Not including a CpG dinucleotide at the most 3’ end of the primer might also result in inefficient or a lack of annealing, and unspecific binding, which could induce both false negative and false positive results [9, 58–61]. Optimal primer/probe length of 20–30 bases and 12–20 bases for minor groove binder (MGB) probes was met in 84.4% of the MSP primers, 69.4% of qMSP primers and 67% of the probes (Fig. 2). An additional 15.6% of the MSP primers, 29.2% of qMSP primers and 33% of probes were suboptimal in length (17–19 or 31–36 bases, < 12 or > 20 bases for MGB probes; Fig. 2). Among the probes suboptimal in length, 87.5% were molecular beacon probes. MGB probes generally allow a shorter probe sequence because of the increase in Tm by the MGB addition, which was accounted for in the results [62]. Molecular beacon probes carry an additional 5–7 bases complementary to each other at the start and end of the sequence, which means these probes are generally longer compared to Taqman probes [63]. In order to take these specific characteristics into account, the primer/probe length criterium was extended to 17–19 bases and to 31–36 bases. Nevertheless, 12.5% of these molecular beacon probes did not comply to the extended primer length criterium. This could potentially lead to inefficient or lack of annealing as well.

Eighty-one percent of MSP primer sets, and 55% of the qMSP primer sets had a similar melting temperature (Tm), meeting the criterium (i.e., Tm forward and reverse primers ≤ 2 °C difference). For the qMSP assays with Taqman probe, 75% of the probes met the criterium (i.e., Tm 5–10 °C higher than the corresponding primer set), and all of the MGB probes met the criterium (i.e., Tm 5–15 °C higher than the corresponding primer set; Fig. 2B). Not adhering to these Tm criteria could again lead to inefficient annealing of (one of) the primers or probe. For probes, an additional criterium was assessed (i.e., no G base at the most 5’ end of the probe), which was met in 95.8% of the included probes. A G base at the most 5’ end of the probe might prematurely quench the fluorophore, resulting in false negative results [64].

Last, optimal amplicon sizes of maximum 120 bp were used in 62.5% of the MSP assays, and 76.7% of the qMSP assays with probe or SYBR. An additional 25% of MSP assays and 13.3% of qMSP assays with probe or SYBR used suboptimal amplicon sizes (121–159 bp), and 12.5% of MSP assays, and 10% of qMSP assays exceeded the acceptable 160 bp amplicon size (Fig. 2). As DNA in liquid biopsies mostly originates from apoptotic and necrotic cells and in this case has to be bisulfite converted, it is highly fragmented with an estimated maximum of ~ 160 bp. However, depending on sample type, cell-free DNA in liquid biopsies can be as small as < 100 bp which should be taken into account when designing an assay [65–69].

Although assay design varied widely, the major criteria to distinguish methylated from unmethylated DNA were covered in most assays. However, several factors should receive additional consideration, such as primer length and Tm (Fig. 2). Probe design factors tend to score poorer compared to primer design factors, and generally, qMSP assays scored worse compared to MSP assays across all criteria (Fig. 2). In addition to the scored items, it is important to adhere to general primer/probe design criteria like a CG content of 30–80% and to ensure that no dimers or hairpin loops form [64, 70–72]. Further, it is important to consider genetic background and to make sure no prevalent single nucleotide polymorphisms (SNPs) appear at the 3’ end of the primer, to allow efficient annealing [73]. Moreover, assays including appropriate controls and a reference gene are most likely to generate reliable results [74].

To measure DNA methylation, several different techniques are currently available for research purposes, of which MSP and qMSP are most widely used. In general, qMSP assays with probe revealed more design flaws compared to both MSP and qMSP with SYBR assays. In the assays with a probe, especially the items regarding probe design showed low scores (Fig. 2). This emphasizes the difficulty of designing qMSP assays where the addition of a probe introduces another layer of complexity to the designing process. However, it can be questioned whether it is a necessity to fully optimize all separate subcomponents of primer and probe design, as assays with suboptimal scores for some criteria may also work. For example, if one of the primers in a set fails to meet the criterium of including ≥ 4–5 non-CpG cytosines per primer, the other primer could compensate, and the assay might work without any problems. This emphasizes that primers and probes should be designed as an assay, rather than single components.

Often, (nested) MSP assays are used in biomarker studies because they require substantially less DNA input compared to qMSP assays. Because of its quantitative nature and the specific binding properties of the utilized probe, qMSP with probe might be preferred over MSP assays for specific research questions. However, qMSP assays with SYBR are prone to false-positive results, as SYBR is an intercalating dye that binds to all double-stranded DNA [75].

After designing an assay, it is advised to perform an in silico analysis of this assay to check for dimers, hairpins and 3’-end primer stability, as extensively described by Davidović et al*.* [58]. In addition, assays should be optimized in terms of PCR conditions, such as PCR component concentrations and annealing temperature, using gradient PCRs. Bisulfite-converted fully methylated, fully unmethylated and no template controls, as well as non-converted DNA and a non-converted no template control should be used in the assay optimization process [76, 77]. Next, pilot studies using small sample sets of interest can evaluate the feasibility of an assay for cancer diagnosis, and minimize false positive and false negative results. Additionally, when analyzing quantitative data, it is important to select an appropriate cutoff value to determine whether a sample is methylated or unmethylated, and several methods to determine the optimal cutoff have previously been postulated [78, 79]. Different cutoffs among studies examining the same assays could, among others, explain the large variation in diagnostic performance, and could therefore hamper comparability of studies [78, 79].

## Conclusions

In this study, using CRC markers as an example, we emphasized the importance of assay design for diagnostic DNA methylation biomarkers, indicating that a rational choice of genomic location and proper primer/probe design upfront are crucial when striving toward a clinically relevant and useful biomarker. This not only applies for diagnostic biomarkers, but for all DNA methylation markers intended to discriminate between two patient categories, such as prognostic and predictive biomarkers. However, only using the recommendations summarized in Box 1 does not guarantee a successful clinically relevant assay. Next to the factors discussed in this article, additional experimental factors can influence the diagnostic performance of DNA methylation biomarkers, such as sample type, quality and composition, assay amplicon size, and bisulfite conversion efficiency [11, 57, 80–85], as well as methodological factors such as sample size, using appropriate controls and statistical analyses. Nevertheless, considering both assay location and assay design upfront could greatly improve future DNA methylation biomarker development and inter-study comparability. To achieve this, future research should focus on linking the technical considerations discussed here to diagnostic parameters and clinical outcome. By optimizing these technical considerations in DNA methylation biomarker development, clinically relevant DNA methylation biomarkers are more likely to be developed.

**Box 1:** Recommendations for DNA methylation assay design

DNA methylation assay design recommendations
*Genomic location* • Before designing a DNA methylation biomarker assay, make a rational choice for the genomic location of the assay • For example, sequencing or publicly available data such as TCGA to identify the optimal genomic location
*Primer- and probe design* • Ensure the primers and probes are able to discriminate unmethylated from methylated DNA • Appropriate amount of CpG dinucleotides and non-CpG cytosines in primers and probe • Ensure the primers and probes have the ability to anneal efficiently • CpG dinucleotides at most 3’ end of primer, primer length, avoiding premature quenching of probe fluorophore • Ensure primers and probes are designed as an assay, rather than single primers and probes • Similar Tm between primers and appropriate Tm of probe relative to the Tm of the primers • Consider sample type in assay development • For liquid biopsies, the total assay amplicon size should be maximum 120 bp
*Assay optimization* • In silico analysis of assay • Optimize PCR conditions • Use appropriate controls • Perform pilot studies • Determine cutoff

## Methods

### Search strategy and study selection

A systematic search until December 2020 was performed in Pubmed, Embase, Cochrane library and Google Scholar, to identify all diagnostic DNA-methylation biomarker studies for CRC. Only original articles in the English language were considered; reviews, editorials and conference abstracts were excluded. Only articles studying DNA methylation through MSP (nested/direct) and qMSP (probe/SYBR), which provided the assay sequences in the article, and studied liquid biopsies (blood, serum, plasma, stool or urine) were included. Articles discussing hereditary cancer syndromes were excluded. From all diagnostic DNA methylation markers for CRC reported in the included studies, eleven were selected for further evaluation (*ALX4*,* APC*,* CDKN2A*,* MGMT*,* MLH1*,* NDRG4*,* SDC2*,* SFRP1*,* SFRP2*,* TFPI1* and *VIM*) as they were described in at least five studies. Diagnostic performance (sensitivity and specificity) was extracted for all genes when available. Although it is one of the most commonly studied biomarkers for early CRC detection, *SEPT9* was excluded due to the fact that most studies (62%) used one of the two commercial assays to measure *SEPT9* methylation.

### Identification optimal genomic location within TCGA data

In order to identify the genomic location where the methylation difference between normal and tumor tissue is the largest, the online available TCGA data visualization tool MEXPRESS [86, 87] was used. TCGA methylation data of the genes of interest in the CRC patient dataset (COAD) were assessed. MEXPRESS visualizes data for specific genes, and all Illumina 450 K methylation array CpGs that have been linked to that gene. All CpGs, irrespective of their location relative to the gene, were assessed. The three locations with the largest methylation difference between normal and tumor tissue (tumor hypermethylated compared to normal in all genes, except for *MGMT*) were extracted and will be referred to as ‘the extracted locations’ throughout this manuscript.

### Primer and probe quality assessment

In order to assess primer and probe quality, two independent observers (M.M. & K.L.) scored all primers and probes according to criteria were constructed based on both literature and expert experience (Table 2). Although we are aware that designing the perfect primers and probes is challenging, and many different criteria have been postulated, we attempted to evaluate the optimal design criteria.

**Table 2:**
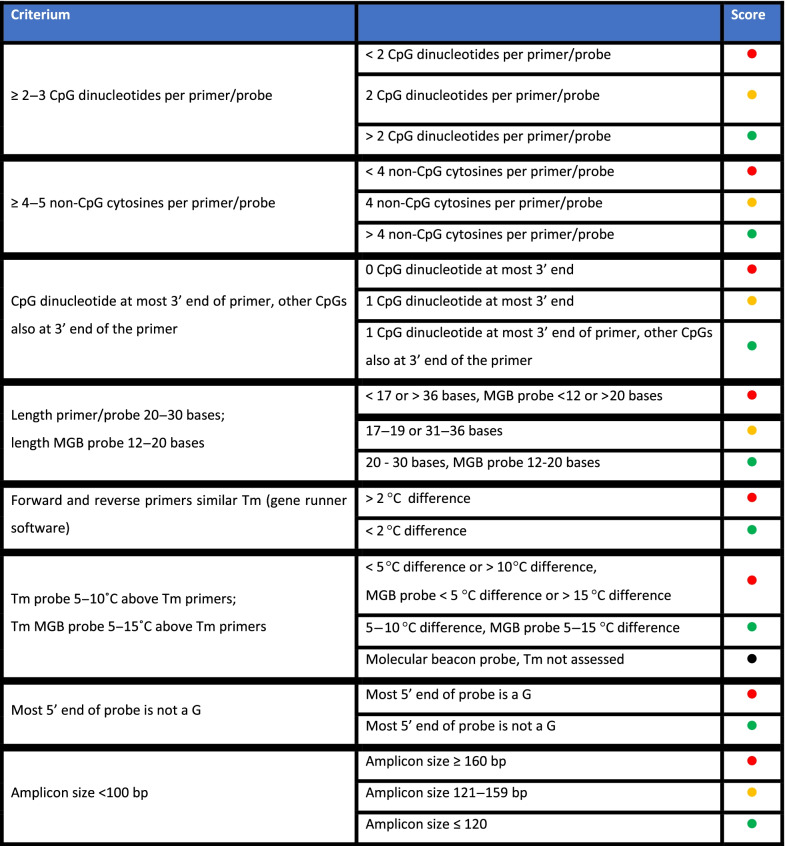
Primer and probe assessment definitions

All criteria apply to (q)MSP primers and probes on the bisulfite-converted sequence of the gene of interest, and to the methylation-specific primer set in case of (q)MSP without probe. As bisulfite-conversion changes unmethylated cytosines to uracil, while methylated cytosines remain unchanged [9], primers and probes should be designed to distinguish methylated from unmethylated DNA and to anneal efficiently. Therefore, at least 2–3 CpG dinucleotides and 4–5 non-CpG cytosines should be included in the primer or probe [9, 58–61]. For optimal annealing, a CpG dinucleotide should be at the most 3’ end of each primer, and preferably the other CpGs are also at the 3’ end of the primer [9, 58–61]. Also, the ideal primer length is 20–30 bases [58], and preferably the forward and reverse primer should have a similar Tm (calculated using Gene Runner software). When using a probe, ideally it is 20–30 bases long, and the Tm is 5–10 °C above the primers’ Tm (calculated using Gene runner software); when using an MGB probe, its length is preferentially 12–20 bases long, and the Tm of MGB probes should preferably be 5–15 °C above the primers’ Tm (calculated using Primer Express software) [64, 72, 88–90]. Additionally, the most 5’ end of the probe cannot be a G, as this might quench the fluorophore [64]. Last, liquid biopsies mostly carry highly fragmented cell-free DNA of maximum ~ 160 bp (length of DNA wrapped around one nucleosome), and DNA is additionally fragmented by bisulfite conversion. Therefore, amplicon size was evaluated, with the preferred amplicon size being a maximum of 120 bp [65–69]. All primers and probes were scored according to these criteria (defined in Table 2). In case a nested approach was used, the inner assay was evaluated. For molecular beacon probes, one of our criteria might not be completely suitable, as (to our knowledge) no design tools exist to calculate the Tm of these probes. Therefore, we were unable to assess the Tm of these probes, and they were specifically marked within Fig. 2. Green dots represent optimal design, orange dots represent suboptimal, but acceptable design. Red dots do not necessarily mean a primer or probe does not work, but rather that there is an increased risk of technical problems with the primer or probe for that specific criterium (Table 2). Black dots mean that the criterium was not assessed for that probe, as it was a molecular beacon probe.

## Data Availability

All data are available in the main text. MEXPRESS is an open source visualization tool for DNA methylation-, expression- and clinical data extracted from TCGA (https://mexpress.be/).

## Abbreviations

CRC: Colorectal cancer
MGB: Minor groove binder
MSP: Methylation-specific polymerase chain reaction
PCR: Polymerase chain reaction
qMSP: Quantitative methylation-specific polymerase chain reaction
Sens: Sensitivity
Spec: Specificity
SNP: Single-nucleotide polymorphism
TCGA: The Cancer Genome Atlas
Tm: Melting temperature
TSS: Transcription start site
